# Forschen in einer extremen Umwelt: Praktiken unterwasserarchäologischer Feldforschung am Kap Gelidonya (1958–1961)

**DOI:** 10.1007/s00048-021-00298-4

**Published:** 2021-05-03

**Authors:** Eike-Christian Heine

**Affiliations:** Institut für Geschichtswissenschaft, Abt. Wissenschafts- und Technikgeschichte, Technische Universität Braunschweig, Schleinitzstr. 13, 38106 Braunschweig, Deutschland

**Keywords:** Honor Frost, Peter Throckmorton, Frédéric Dumas, Schwammtaucher, Unterwasserarchäologie, Feldforschung, Honor Frost, Peter Throckmorton, Frédéric Dumas, Sponge divers, Underwater archaeology, Field studies

## Abstract

1958 startete vor der kleinasiatischen Küste eines der ersten Vorhaben für eine systematische archäologische Kampagne unter der Wasseroberfläche. Dabei wurde vor dem Kap Gelidonya das Wrack eines bronzezeitlichen Schiffs untersucht. Um die Arbeitsweise und die konkreten Probleme der Unterwasserarchäologie näher zu beleuchten, umfasst dieser Beitrag neben einer Beschreibung der konkreten Forschungsgegenstände und der eingesetzten technischen Hilfsmitteln auch die naturräumlichen Eigenschaften sowie die politischen Rahmenbedingungen jener Zeit. Am Übergang zwischen kolonialem Zeitalter und Kaltem Krieg zeichnet er zudem die biografischen Wege nach, welche die zentralen Akteur*innen der Kampagne am Fundort zusammenführten. Das ist notwendig, um zu verstehen, wieso Taucher*innen, die keine etablierten Archäolog*innen waren, diese Kampagne anstießen und durchführten. Der Artikel analysiert außerdem den Stand der Forschung im Jahr 1960 in diesem Feld und die Schwierigkeiten und Lösungen bei der Arbeit unter Wasser. Er versteht sich als Beitrag zur noch ungeschriebenen Geschichte der Unterwasserarchäologie.

## Abstract (en)

In 1958, one of the first projects to carry out a systematic archaeological campaign underwater began off the coast of Asia Minor. At Cape Gelidonya, the wreck of a Bronze Age ship was investigated. To understand the concrete problems and working methods of underwater archaeology, it is important to account not only for the tangible objects of research and the technical aids used, but also the natural features and the political framework in which actors operated. Situated in the years between the end of the colonial era and the Cold War, this article traces the biographical paths that brought together central actors at the site of the discovery. This perspective is necessary for understanding why divers who were not established archaeologists initiated and carried out the campaign. The article also analyzes the research of the campaign carried out 1960 and explores some of the difficulties and solutions encountered in working underwater. The article is a contribution to the still unwritten history of underwater archaeology.

## Das Wrack von Kap Gelidonya

Im Jahr 1958 wurde vor der südtürkischen Küste eine der ersten systematischen archäologischen Kampagnen unter der Wasseroberfläche in Angriff genommen. Dabei wurde ein Schiffswrack untersucht, das im 14. Jahrhundert v. Chr. am Kap Gelidonya gesunken war und Metallbarren sowie mykenische Töpferware geladen hatte. Der vorliegende Aufsatz nimmt die Umstände und Arbeitsmethoden bei diesem frühen unterwasserarchäologischen Experiment genauer in den Blick.

Den Praktiken der Feldforschung – verstanden als die Frage nach den Gegenständen, Methoden und Ursachen der Forschung – unter Wasser nähere ich mich wie folgt an: Zunächst konzentriere ich mich auf die zentralen Akteur*innen der Kampagne, die allesamt Außenseiter*innen der akademischen Archäologie waren. Der US-Amerikaner Peter Throckmorton (1928–1990), die Britin Honor Frost (1917–2010), einige Schwammtaucher aus Bodrum und der Franzose Frédéric Dumas (1913–1991) brachten unterschiedliche Interessen und Fähigkeiten ein, die für das Verständnis der Erforschung des Wracks von Kap Gelidonya wesentlich sind. Die folgenden Abschnitte berichten vom Beginn der ersten Kampagne im Sommer 1960, den dabei anzutreffenden Hindernissen und Lösungen bei der Arbeit unter Wasser sowie vom zentralen Erkenntnisinteresse der Taucher*innen. Das Fazit ordnet die Ergebnisse dieses Grabungsjahres vor dem Hintergrund der sich formierenden Fachrichtung Unterwasserarchäologie ein und zeigt, inwieweit die politischen Rahmenbedingungen jener Zeit zwischen Dekolonialisierung und frühem Kalten Krieg grundlegend für die Forschungspraktiken waren.

**Figure 1:**
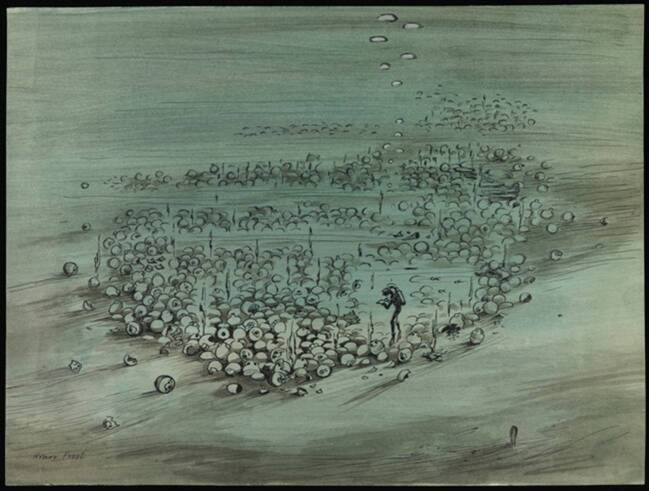

Thesenhaft zugespitzt war das Ziel der Unterwasserarchäolog*innen erstens, Maximen der zeitgenössischen Feldforschung in einem neuen Naturraum umzusetzen. Angestrebt wurde neben der Sicherung der Fundstücke vor allem die genaue Vermessung und präzise Dokumentation der Fundstätte, wobei die Repräsentation des Fundortes in Karten zum größten Problem wurde. Zweitens war die für das Vorhaben zentrale Technik das Gerätetauchen mit Druckluftflasche, Mundstück und Taucherbrille, das nach dem Zweiten Weltkrieg zu einer Popularisierung des Tauchens geführt hatte. Damit gingen bestimmte Rahmenbedingungen einher, die Einfluss auf den Forschungsalltag hatten – etwa eine begrenzte Tauchdauer oder die medizinische Notwendigkeit, nur zwei Tauchgänge pro Tag durchführen zu können. Zugleich probierten die Forscher*innen neue Techniken aus und modifizierten sie gegebenenfalls, sodass sich die Feldarbeit hier als eine Bricolage darstellt, bei der sich Forschungstechniken und Forschungsfragen voneinander abhängig zeigen. Drittens zeige ich, dass zwei Umstände mit in den Blick genommen werden müssen, um die konkrete Ausgestaltung der Feldforschung zu verstehen. Das sind einerseits die naturräumlichen Eigenschaften der Unterwasserwelt, die eine extreme und lebensgefährliche Umwelt darstellt: Strömung und Sedimente formen Fundorte um, das Wasser sorgt für visuelle Verzerrungen, der Druck in der Tiefe verändert die Wahrnehmung der tauchenden Forscher*innen und bedroht ihre Gesundheit. Die Welt unter der Oberfläche ist für die enthusiastischen Taucher*innen, die mit ihren technisierten Körpern in die Tiefe steigen, ein Ort des modernen Wunders (Adamowsky 2017) (Abb. 1). Zugleich ist sie ein Naturraum, in dem wissenschaftliche Erforschung und technische Erschließung nicht voneinander zu trennen sind. Viertens mache ich deutlich, dass die Kampagne am Kap Gelidonya von gewissen politischen Rahmenbedingungen geprägt war.

Zur Geschichte der Ozeane wurde in den letzten Jahren intensiv geforscht (vgl. Rozwadowski 2018; Gillis & Torma 2015). Zur Geschichte des Tauchens hingegen liegen bislang überraschend wenige Arbeiten vor, die kulturgeschichtliche Perspektiven der Wissens-, Technik- oder Umweltgeschichte einnehmen (Rozwadowski 2012). Die Geschichte der Unterwasserarchäologie wiederum wird bislang nur in Selbstzeugnissen von Forscher*innen (vgl. im Kontext des Aufsatzes Frost 1963; Throckmorton 1964; Bass 1966) und in chronologischen Abrissen von Pionierprojekten greifbar.^1^ Indem die vorliegende Studie an Arbeiten anknüpft, die sich mit der Geschichte der Feldwissenschaften auseinandersetzen (vgl. Vetter 2016), eröffne ich neue historische Perspektiven auf die Unterwasserarchäologie und liefere einen Beitrag zur Geschichte der Erforschung der Meere in der zweiten Hälfte des 20. Jahrhunderts.

Zudem knüpft der Beitrag an die Frage an, wie sich das Fach Archäologie im Zusammenhang mit neuen Techniken wie der Fotografie oder der DNA-Analyse verändert hat (vgl. Brusius 2015; Bösl 2017). Stefanie Klamms Arbeit über Visualisierungstechniken der Landarchäologie im 19. Jahrhundert erlaubt eine Einordnung der hier vorgestellten Ergebnisse in die Geschichte der Archäologie. So ist die Kampagne am Kap Gelidonya ein Beispiel, bei dem Karten als Methoden der Darstellung zentral waren. Auch im Untersuchungsfall war „die Beziehung der Archäologie zu ihren Medien von besonderer Art“, weil diese Medien – das heißt hier die Karten – ein „wesentliches […] Arbeitsmittel der Archäologie“ waren, um die Aneignung jener Strukturen zu erlauben, die „im Prozess des Ausgrabens […] verändert und zerstört“ wurden (Klamm 2017: 17).

Beim unterwasserarchäologischen Vorhaben am Kap Gelidonya wird zudem das „bewegliche […] Verhältnis zwischen epistemischen und technischen Momenten im Forschungsprozess“ deutlich, das Hans-Jörg Rheinberger anspricht (Rheinberger 2006: 31). Um die Forschungspraxis zu beschreiben – also den „Entdeckungszusammenhang“ (ebd.: 27) in der Feldarbeit nachzuzeichnen –, beziehe ich mich auf Begrifflichkeiten, die für den Laborbereich entwickelt wurden. Für die Erforschung der Unterwasserwelt ist umfangreiches technisches Equipment notwendig, angefangen bei der Tauchausrüstung. Insbesondere in Abschnitt 6 zeige ich auf, dass bei dieser Mission viel improvisiert wurde, um in der „flüssigen Umwelt“ verlässliche Daten zu produzieren. Hier wird auch deutlich, wie eng die Epistemologie – also jene „Dinge, denen die Anstrengung des Wissens gilt“ (ebd.: 27) – mit den Techniken der Forschung unter der Wasseroberfläche verschränkt war.

Der vorliegende Aufsatz ist vor allem durch das 2018 zugänglich gemachte Honor-Frost-Archiv an der University of Southampton möglich geworden.^2^

Neben Frosts Korrespondenz und ihren Notizen stellen publizierte Grabungsberichte und Erinnerungen von Teilnehmer*innen der Kampagne die wichtigsten Quellen dar.

Mein Beitrag ist dennoch lediglich ein erster Schritt in einer wissenschaftshistorischen Untersuchung der Formierung der Unterwasserarchäologie als Disziplin in der zweiten Hälfte des 20. Jahrhunderts. Weitere Forschung wird notwendig sein, um die hier vorgestellten Ergebnisse historisch einordnen zu können. Einen Ausgangspunkt bieten etwa Überblicke über wichtige Kampagnen, Akteure und Institutionen (Mainberger & Weski 2014; Meide 2013).

Der gewählte Fokus auf Forschungspraktiken zeigt schließlich am Beispiel der Unterwasserarchäologie auch, wie die Erforschung extremer Umwelten im 20. Jahrhundert mit der Entwicklung von Überlebenstechniken und anderen technischen Lösungen verschränkt war. Während der vorliegende Aufsatz als Fallstudie zwar auf die konkreten Ereignisse vor der kleinasiatischen Küste fokussiert, eröffnet sich doch eine größere Schnittstelle der Wissenschafts-, Technik- und Umweltgeschichte, die sich etwa zur selben Zeit und in ähnlicher Weise im Hochgebirge oder in der Polarforschung zeigt (Rozwadowski 2010a; Clements 2018; Herzberg et al. 2018: 3). Der vorliegende Beitrag zur Unterwasserarchäologie schließt so auch an eine Geschichte der Feldwissenschaften und der Expeditionen an, die Fragen der Global- und Zeitgeschichte aufwirft und unter anderem darum kreist, wo die Formierungen jener Verhältnisse von Wissen, Technik und Umwelt anzusetzen sind, welche Umweltzeitalter und Anthropozän prägen.

## Peter Throckmorton und das Erbe des kolonialen Zeitalters

Ein Initiator der Untersuchungen vor dem Kap Gelidonya war Peter Throckmorton. Seine Beschäftigung mit Wracks am Boden des Mittelmeers ergab sich aus verschiedenen Ereignissen in seinem Leben, die er selbst in der Tradition des abenteuerlichen kolonialen Reiseberichts schildert. Throckmorton hatte sich von Indien aus auf dem Landweg nach Westen aufgemacht: „In the spring of 1958 four of us were driving across Afghanistan toward Europe […]. We had been in Central Asia for a month […]. Our insides ached with dysentery […]. We were tired of each other and we stank.“ (Throckmorton 1964: 8) Der Bericht über die Unterwassergrabungen beginnt bei Herat, wo der Wagen seiner Reisegruppe im Sand stecken blieb. Während noch versucht wurde, das Gefährt zu befreien, zog ein Sandsturm auf: „In the middle of it all the wind began to howl and a yellow cloud covered the sun […]. We crawled onto the car and tried to breathe, while the sand blasted the closed windows.“ Anekdotisch verweist der Reisebericht an dieser Stelle erstmals auf den Sehnsuchtsort und das Ziel der Reise: Ein Mitreisender „whispered softly: The sea, Peter, do you remember the sea?“. Throckmorton entwirft in seiner Antwort ein Bild des Meeres als „Hort von Wundern“ (Adamowsky 2017: 11): „For a moment I held back, and then gave myself up to the old dream of the cool and faultless sea. We talked of coral gardens, the habits of sharks […], and of wrecks.“ (Throckmorton 1964: 9).

Nachdem Throckmorton ein altes Motiv des wissenschaftlichen Reiseberichts im Orient aufgreift – nämlich das mehr oder weniger heroische Überwinden einer unzivilisierten Natur –, folgt im nächsten Absatz ein weiteres Stereotyp: der Verweis auf die Antike, mit dem der neuzeitliche Autor sich in die klassische Tradition einschreibt. Throckmorton bemüht den griechischen Politiker, Feldherrn und Schriftsteller Xenophon, der an der Wende zum 4. Jahrhundert v. Chr. in die kriegerischen Auseinandersetzungen zwischen Griechen und Perser verstrickt war. Nachdem der griechische Anführer Kyros bei einer Expedition in Kleinasien gefallen war, schlug sich Xenophon mit anderen Überlebenden wieder zum Mittelmeer durch. Seine eigene Ankunft am Mittelmeer inszeniert Throckmorton ein zweites Mal, nun in diesem antiken Bericht gespiegelt: „Eventually we came over the pass in the mountains of Trebozid, where Xenophon, fearing another attack by the natives, galloped to the head of the pass to discover his men shouting: ‚The sea, the sea.‘“ (Throckmorton 1964: 9).

In der Türkei angekommen, treten die Topoi des kolonialen Zeitalters zunächst in den Hintergrund. Throckmorton entdeckte die Unterwasserarchäologie und die Unterwasserfotografie als neue Betätigungsfelder; beides ließ sich aufs Beste mit seiner Leidenschaft für das Meer verbinden. In Istanbul lernte er Mustafa Kapkin kennen, der Fotograf sowie Mitglied im Tauchclub von Izmir war. Mit ihm machte er sich auf zur kleinasiatischen Küste. Kapkin wurde für Throckmorton ein „invaluable partner and ally“, der ihn mit der Begeisterung für das Tauchen an antiken Wracks ansteckte. „At thirty-seven he was one of the best photographers in Turkey, and the first Turk to make underwater photographs.“ (Throckmorton 1964: 10–11) Nachdem die beiden in einigen Tauchgängen antike Amphoren geborgen hatten, stellte Kapkin Throckmorton einen Freund vor: den Direktor des Antikenmuseums von Izmir, Hakki Gültekin. Gemeinsam mit ihm sei der Entschluss gefasst worden, den Berichten lokaler Fischer nachzugehen und sich an einer archäologischen Erkundung unter Wasser zu versuchen.

Einige Wochen später kamen Kapkin und Throckmorton in Bodrum an, dem antiken Halikarnassos. Hier sah sich Throckmorton an seine Familiengeschichte und damit erneut an das Zeitalter des Kolonialismus erinnert. Niemand Geringeres als Sir Walter Raleigh, Pirat und Umsetzer kolonialer Projekte ihrer Majestät Elisabeth I., hatte nämlich seine Vorfahrin Bess Throckmorton geheiratet. Peters Familienzweig war später nach Virgina übergesiedelt. In der verfallenen Festung über dem Hafen von Bodrum, die von Kreuzfahrern aus Überresten des Mausoleums – einem der sieben Weltwunder – erbaut worden war, habe Throckmorton das Wappen seiner Vorfahren entdeckt (Frost 1990: 181). In seinem letzten Buch aus dem Jahr 1987 notiert Throckmorton über seine Ankunft in der alten Hafenstadt: „I had a weird feeling I had been there before – as the 11th generation descendant of the man who raised funds to build the castle I was moved by its evocative ruin and determined to see it restored.“ (Throckmorton 1987: 13).

Solche Verweise auf das koloniale Zeitalter blieben von den anderen Teilnehmer*innen der Kampagne nicht unkommentiert: „Not everyone ‚determines‘ to restore a castle.“ (Frost 1990: 191–192) In einem Nachruf kommentiert Frost dieses Selbstbewusstsein, bevor sie sich mit Throckmortons Neigung zur „Kolonialherrenart“ kritisch auseinandersetzt. Auch wenn er kein reicher Mann gewesen sei, so sei er doch großzügig gewesen, habe Funde stets für die Nachwelt aufbewahrt und die Unterwasserarchäologie aus eigenen Mitteln finanziert: „His vision was seldom at fault and his intentions always personally disinterested and financially genereous.“ Bemerkenswert für einen Nachruf ist dann folgende Äußerung Frosts: „Nevertheless, the adage ‚nanny knows best‘ coming from a member of a super power, is as aggravating to members of lesser states as it is to small children in the nursery […] and indeed to grown ups“. Offensichtlich war das Verhältnis der beiden nicht ganz ungetrübt. Throckmortons Schriften und sein Verhalten in Bodrum weckten bei Frost zudem Erinnerungen an das koloniale Zeitalter: „In modern jargon his stance could be misinterpreted as being ‚imperialistic‘ […]. [A] charge that would be doubly false, for Peter was neither a ‚modern man‘ nor a ‚political animal‘.“ (Ebd.).

## Honor Frost: Tauchen und Archäologie

Als Honor Frost 1958 in Bodrum ankam, war sie bereits eine erfahrene Taucherin und hatte sich praktische Kenntnisse in der archäologischen Feldforschung angeeignet. Aber auch sie war keine studierte Archäologin. Nach dem frühen Tod ihrer Eltern von dem reichen Londoner Kunstsammler Wilfried Ariel Evill großgezogen, hatte sie seit 1954 viel Zeit an der Französischen Riviera verbracht und dort das Gerätetauchen erlernt.^3^ Diese neue Technik mit Maske, Mundstück und Druckluftflasche war während des Zweiten Weltkriegs erfunden worden und hatte – dank ihrer Einfachheit und Sicherheit – Tauchen in der Nachkriegszeit zum Breitensport gemacht. Frost arbeitete als Tauchlehrerin in Cannes und verbrachte dort auch einen Großteil ihrer Freizeit unter Wasser (Frost 1963: 4–18). In den Monaten vor ihrer Ankunft in Bodrum hatte sie erste Erfahrungen in der Feldarbeit gesammelt, indem sie bei Kathleen Kenyons Ausgrabungen in Jericho wie auch bei den Kampagnen des Institut français d’Archéologie de Beyrouth und des Département des Antiquités de Syrie mitwirkte (Pomey 2019: 11–12). Frost verstand sich nach diesen Erfahrungen als *draughtsman *(dt. Zeichner*in). Der Zugang zur archäologischen Feldarbeit war ihr durch ihr künstlerisches Talent und wohl auch durch ihre guten Verbindungen möglich geworden. Frost hatte vor dem Zweiten Weltkrieg Kunst und Design studiert und anschließend Bühnenbilder fürs Ballett entworfen sowie Ausstellungskataloge für die Tate Gallery betreut (Basch 2019).

Throckmorton erinnert sich in seinen Aufzeichnungen an das erste Zusammentreffen mit Frost, nachdem sie in Bodrum angekommen war, wo er und Kapkin gerade ihre Pläne zu einer archäologischen Unterwassergrabung vorantrieben. Als er gerade im Postamt Tee trank, sei ein Junge hereingestürmt und habe verkündet, dass Amerikaner*innen in der Stadt seien. Diese hätten sich bald als Engländer*innen herausgestellt, und zu seiner Freude sei Honor Frost mit ihrem Zeichentalent unter den Neuankömmlingen gewesen. Anders als er selbst habe Frost Erfahrung in archäologischer Feldarbeit: „[She] had worked for one of the most thorough of modern archaeologists, Miss Kathleen Kenyon […]. Furthermore, she was a handsome blonde.“ Im Verlauf ihrer ersten Unterhaltung habe sie bemerkt: „‚I’ve brought my valve and bottle.‘ I replied that that was nice, and that raki did in fact get a bit tiresome, then did a doubletake and realized that she meant aqualung and tank.“ Ihm wurde somit klar, dass sie auch eine Taucherin war: „She had been absorbed by the idea of developing underwater drafting techniques ever since she had worked for Miss Kenyon. She was provided with a small British aqualung, an alcohol stove, a thick notebook, a red bathing suit, a packet of China tea, and a lot of luck […].“ Throckmorton war begeistert: „I have seldom been so happy to see anybody.“ (Throckmorton 1964: 62–64).

Frost erinnert sich an dieses erste Zusammentreffen deutlich anders. Tatsächlich sei sie in die Türkei gekommen, um im Museum von Izmir eine aus dem Mittelmeer gehobene Statue der Demeter zu besichtigen. Von dort habe man sie nach Bodrum geschickt, wo zwei Mitglieder des Tauchclubs von Izmir gerade auf der Jagd nach antiken Wracks seien. Auf der Suche nach diesen türkischen Tauch- und Antikenenthusiasten habe sie schließlich nicht nur Kapkin gefunden, sondern sei auch zufällig auf Throckmorton gestoßen, über den sie notierte: „Geographically and metaphysically, we reached the same point from different directions.“ Er habe die türkischen Wracks zufällig – und nicht zielgerichtet wie sie – entdeckt. „Being the first ancient wrecks he had seen, they came as a revelation, and Peter was not one to dismiss revelations.“ Throckmorton erschien ihr ebenso enthusiastisch wie eigenwillig. Er habe sich in die Zivilisation des Mittelmeers verliebt. Seine anthropologische Perspektive unterscheide sich allerdings deutlich von ihrer eigenen: „In matter of theory, our views were bound to differ, but in practice we all worked together happily.“ Trotzdem war auch aus ihrer Sicht das zufällige Treffen mit Kapkin und Throckmorton im Hafencafé von Bodrum bedeutsam: „The upshot of our meeting was that we left together, as Kpt. Kemal’s guest […] to look at some wrecks.“ (Frost 1963: 151–154).

## Schwammtaucher und Unterwasserarchäologie

„The Innocent Adventurers“, wie Throckmorton die Tauch- und Unterwasserenthusiast*innen um sich herum nannte, konnten überhaupt nur mit der Suche nach Wracks beginnen, weil es in Bodrum Ende der 1950er-Jahre noch Schwammtaucher wie Kapitän Kemal gab. Die Geschichte dieses Berufs muss zu großen Teilen noch geschrieben werden.^4^ Für die Unterwasserarchäologie waren die Schwammtaucher von zentraler Bedeutung. Bevor Kunststoff als fast universell einsetzbares Material in den Haushalt einzog, suchten diese Apnoetaucher schon seit Jahrhunderten oder gar Jahrtausenden den Boden des Mittelmeers nach Naturschwämmen ab. *Meyers Konversations-Lexikon* notierte 1885: „Im Griechischen Meer und an der syrischen Küste gewinnt man den Badeschwamm von Mai bis Ende September durch Taucher. Sie gehen 18 m tief und halten 90 Sekunden bis 3 Minuten aus.“^5^ Das Zentrum der Schwammtaucher waren die Ägäis, die griechischen Inseln und die kleinasiatische Küste. Um 1900 stachen von hier rund 250 Schiffe in See und befuhren das gesamte Mittelmeer. Als im 19. Jahrhundert die Schwämme durch die intensive Ernte immer seltener wurden und zugleich die Nachfrage anzog, suchte man nach Ersatz – und fand ihn beispielsweise in Form des Schwammkürbisses oder aus Zellstoff hergestellter Schwämme. Gleichzeitig bezog man die Meerschwämme nicht mehr allein aus dem Mittelmeer, sondern importierte sie auch aus der Karibik oder dem Indischen Ozean.^6^ Die Fischer des Mittelmeers versuchten ihrerseits, durch technische Innovationen den Ertrag zu steigern. Um größere Tauchtiefen zu erreichen und bei einem Tauchgang länger unter Wasser bleiben zu können, stellten sie um 1900 auf ein neues Tauchsystem um. Bei der hier eingesetzten Form des Helmtauchens ist ein Bronzehelm fest mit einem Anzug verbunden. Ein Kompressor an Bord des Schiffes sichert über einen Schlauch die Luftversorgung. Im Helm muss in der Regel ein leichter Überdruck erzielt werden; den Ausgleich zum Tiefendruck stellt ein Ventil sicher. Der vom Kompressor erzeugte Druck muss also relativ genau geregelt werden, was nicht ganz ungefährlich ist (Möser 1992: 194–195).

Für die frühe Phase der Unterwasserarchäologie in der ersten Hälfte des 20. Jahrhunderts waren diese Schwammtaucher entscheidend. Eine frühe Bergung der Ladung eines antiken Wracks hatte 1901 bei Antikythera stattgefunden. Hier holten Schwammtaucher spektakuläre Bronze- und Marmorstatuen aus der Tiefe, aber auch Tonlampen, Amphoren und andere Alltagsgegenstände. Die griechischen Behörden waren zufällig auf diese Funde aufmerksam geworden und organisierten und finanzierten die Hebung weiterer Artefakte. Dabei kam der „Mechanismus von Antikythera“ ans Licht – ein analoger Rechner aus der Zeit um 70 v. Chr., der verschiedene Kalender parallel ausgibt und so filigran gearbeitet war, wie man es aus der Antike bis dato nicht kannte (Jones 2017: 5–15, 233–245). Das zweite wichtige unterwasserarchäologische Projekt war die Bergung eines Wracks vor Mahdia gewesen. 1907 bestätigte Alfred Merlin (1876–1965), der Direktor der Antikenverwaltung in Tunis, dass es sich bei den Funden, die Schwammtaucher aus der Ägäis vor der tunesischen Küste gemacht hatten, tatsächlich um antike Bronzestatuen handelte. Mit Finanzierung der Académie des inscriptions et belles-lettres und mit Unterstützung durch die Marine bargen griechische Schwammtaucher in den Sommern bis 1913 große Teile der Ladung des im 1. Jahrhundert v. Chr. versunkenen Schiffs. Die Ladung stammte wahrscheinlich aus Athener Werkstätten und war für die reiche römische Oberschicht im südlichen Mittelmeer gedacht. Neben Bronzefiguren und -objekten fand man auch große architektonische Marmorelemente (Hellenkemper Salies 1994: 5–9).^7^

Beide Bergungsoperationen waren charakteristisch für die Anfänge der Unterwasserarchäologie in der ersten Hälfte des 20. Jahrhunderts. Schwammtaucher hatten die Fundstellen jeweils entdeckt und besorgten den Großteil der Bergung; Fundzusammenhänge wurden keine festgestellt und auch die fragilen Überreste der Schiffe selbst fanden kaum Erwähnung in den Publikationen. Stattdessen wurde nur die Ladung der Wracks gehoben, was jedoch zumindest wichtige Artefakte und damit einhergehend Erkenntnisse über Handelsströme ans Tageslicht brachte. Diese Praxis wollten Throckmorton und vor allem Frost ändern. Ihr Vorhaben war, Grabungspraktiken für die Unterwasserarchäologie zu entwickeln, die an die Standards archäologischer Feldarbeit auf dem Festland anknüpften. 1958 blieben Throckmorton und Frost nur noch wenige gemeinsame Tage, bevor sie nach New York respektive London zurückkehren mussten. Sie nutzten diese Zeit, um sich zusammen mit Kapkin einige Wracks zeigen zu lassen. Im Sommer 1959 trafen sich Throckmorton und Frost dann erneut in Bodrum. Nun nahmen sie eine genauere Untersuchung zweier Wracks vor, die ihnen vielversprechend erschienen, erstellten erste Übersichtskarten der Fundplätze und nahmen Proben. Anschließend fuhren sie wieder in ihre Heimat, um Unterstützer*innen für ihr Vorhaben einer systematischen Grabung zu finden.

## Frédéric Dumas, das Wrack von Grand Congloué und neue Ansprüche an die Archäologie unter Wasser

Den ersten Erfolg konnten sie feiern, als Throckmorton im Herbst 1959 Frédéric Dumas für das Vorhaben gewann. Dumas war einer der erfahrensten Taucher der Welt und ein Star der Tauchszene an der Mittelmeerküste. Er, Philippe Tailliez und Jacques-Yves Cousteau waren unter einem Spitznamen bekannt, der an Alexandre Dumas’ Abenteuerhelden um d’Artagnan erinnert: „Les Trois Mousquemers“ (Norton 1999: 221). Frédéric Dumas wurde 1913 geboren und zog im Alter von sechs Jahren mit seiner Familie in das Fischerdorf Sanary-sur-Mer.

Dumas war einer der ältesten Freunde und Mitarbeiter von Cousteau.^8^ Gemeinsam mit anderen zogen sie sich nach dem zwischen Frankreich und dem Deutschen Reich vereinbarten Waffenstillstand vom 22. Juli 1940 in ein Haus an der Mittelmeerküste zurück. Zunächst im unbesetzten Frankreich, nach dem Einmarsch der Wehrmacht in Südfrankreich im November 1942 auch während der Besatzung, verbrachte die Gruppe um Cousteau ihre Zeit mit Tauchen, Fischen sowie damit, Tauch- und Kameratechniken zu erlernen (Madsen 1986: 53; Cousteau & Dumas 1953: 54). 1943 meldete Cousteau gemeinsam mit Émile Gagnan, einem Ingenieur von Air Liquide, ein Patent für eine neue Tauchtechnik an. Herzstück dieses „Scuba“ oder „Aqualung“ genannten Systems ist – neben Taucherbrille, Mundstück und Gasflasche – ein Ventil, das den Druck des hochkomprimierten Atemgases in der Flasche automatisch auf den Umgebungsdruck absenkt. Das System vereinfachte das Tauchen enorm. Auf den massiven Helm konnte nun ebenso verzichtet werden wie auf den Schlauch, der den Taucher an den Kompressor fesselte und dessen Beschädigung sofort Lebensgefahr bedeutete. Beim Helmtauchen sank der/die Taucher*in von Gewichten beschwert auf den Meeresgrund ab und ging schwerfällig auf diesem entlang. Das Gerätetauchen erlaubte hingegen die freie Bewegung im Wasser, was viele Taucher*innen euphorisch schilderten (Rozwadowski 2010b: 162–164; Cousteau & Dumas 1953: 20). 1945 ließen sich Cousteau und Gagnan ihre Aqualunge für den Export patentieren, und eine Tochterfirma von Air Liquide sorgte für die weltweite Vermarktung (Madsen 1986: 37–52).

Dumas war nicht nur an den ersten Tauchgängen, den Film- und Unterwasserexperimenten, sondern auch an Cousteaus Filmen beteiligt. Nachdem sich 1942 die französische Flotte durch Selbstversenkung der Beschlagnahmung durch Deutschland entzogen hatte, unterstütze Cousteaus Equipe die Räumungsarbeiten. Die bei diesen Gelegenheiten entstandenen Aufnahmen verwendeten sie für den 1943 veröffentlichten Dokumentarfilm *Épaves* („Wracks“), bei dem auch Dumas als tauchender Darsteller auftrat. Cousteau notierte dazu: „Wir liebten es, der Lebensgeschichte der Schiffe, ehe sie in die Tiefe absanken, nachzugehen.“ (Cousteau & Dumas 1953: 54) Cousteaus weltweiter Durchbruch war der Film *Die schweigende Welt*, der 1956 sowohl eine Goldene Palme als auch einen Oscar gewann. Dumas trat nicht nur erneut in diesem Film auf, sondern war auch Co-Autor der dazugehörigen reich bebilderten Publikation.

Am 25. November 1959 schrieb Dumas seinen ersten Brief an Frost. Throckmorton habe ihn gefragt, ob er nicht bei der Bergung eines der Wracks vor Bodrum helfen könne. Er habe begeistert zugesagt: „Although I am dealing with many items underwater, archaeology fascinates me and I have been lucky enough to dive at Mahdia [and] Antikythera […]. I worked with Cousteau on the ship of Grand Congloué.“^9^ Die Ausgrabung vor Le Grand Congloué, einer Insel bei Marseille, gilt als die erste Kampagne „to be excavated exclusively by modern methods“, wie Frost kommentiert (Frost 1963: 232). Mit dem neuen Scuba-Tauchsystem, neuartigen Ausgrabungstechniken und der Inszenierung der Ausgrabung als Medienereignis stellt das Projekt einen wichtigen unterwasserarchäologischen Bezugspunkt dar (vgl. Delgado 1997: 174–175).

Cousteaus Bergungsmannschaften setzten beim Wrack von Grand Congloué auf den Einsatz technischer Neuerungen, die eigens dafür entwickelt worden waren. Am wichtigsten war eine Vorrichtung, die Schlamm vom Meeresboden an die Oberfläche transportierte. Das Maschinenhaus dieses *sucaseuse* genannten Systems wurde mithilfe der Marine auf dem Riff eingerichtet, an dessen Fuß ein Wrack mit Hunderten von Amphoren lag. Der mächtige Staubsauger konnte über einen Ausleger über das Wasser geführt werden und bestand aus einem flexiblen metallischen Schlauch, der dem Tiefendruck widerstehen konnte und dessen Ende Taucher*innen lenkten. Komprimierte Luft wurde durch eine Leitung in die Nähe der Schlauchöffnung gebracht und strömte im Schlauch nach oben. Der entstehende Unterdruck riss Feststoffe mit sich und drückte die *émulsion* an die Oberfläche. Das andere Ende des Schlauchs öffnete sich in einem Filter, der fern des Wracks positioniert wurde, sodass das aufgesaugte Material nicht wieder auf die Grabungsstelle zurückfiel. „So brutal diese Methode des Absaugens auch ist“, notierte der Archäologe Fernand Benoit aus dem Marseiller Archäologiemuseum im Château Borély, der an Deck von Cousteaus Schiff „Calypso“ die Funde entgegennahm, „sie hat den Vorteil, dass sich im Korbfilter […] die kleinen Objekte und Fragmente aus Töpferwaren“ sammeln. Diese Auffangeinrichtung sei „das Sieb der oberirdischen Ausgrabung, das unter Wasser unmöglich anzuwenden ist“. Benoit spricht von der *sucaseuse* als einer „doppelt ergänzenden Technik“, die vergleichbar mit Hacke und Kratzeisen bei terrestrischen Grabungen das „minutiöse Abräumen eines speziellen reichen Abschnitts“ gestatte (Benoit 1961: 22).

Gemeinsam mit dem französischen Fernsehen und einem englischen Kamerahersteller inszenierte Cousteau eine Live-Fernsehübertragung aus der Unterwasserwelt: Vor den Augen der Öffentlichkeit wurde ein Stück Kiel gehoben (Benoit 1961: 12). Dank der intensiven Berichterstattung, zu der auch zahlreiche, mit beeindruckenden Fotos bestückte Presseberichte – etwa im *National Geographic* – gehörten, war die Grabung vor Le Grand Congloué bald Archäolog*innen, Tauchenthusiast*innen und der Öffentlichkeit gleichermaßen bekannt (Cousteau 1952, 1954).

Nachdem Dumas Frost in seinem Brief im November 1959 seine unterwasserarchäologischen Erfahrungen geschildert hatte – die mit Mahdia, Antikythera und Grand Congloué die bekanntesten Fundorte der letzten fünfzig Jahre umfassten –, bedauerte er den Stand der Forschung: „Unfortunately, professional archaeologists are much more interested by the cargo than by the ship. I hope we will find time to discuss the matter.“^10^ Wie Throckmorton und Frost war er also daran interessiert, wissenschaftliche Methoden der Unterwassergrabung zu entwickeln und über das verbreitetet „Grab and Run“ hinauszugehen. Zeit, ihre Ideen zu diskutieren, fanden Dumas und Frost bereits wenige Wochen später – am 19. Dezember reiste Frost zu ihm nach Sanary-sur-Mer. Was sie über die Planung der Unterwassergrabungen vor Bodrum hinaus motiviert hatte, Dumas’ Einladung so schnell nachzukommen, war die Aussicht, durch diesen neuen Kontakt selbst zum Wrack von Grand Congloué hinabtauchen zu können.

Frost war sehr erfreut, als sie das berühmte Wrack tatsächlich besichtigen konnte: „It is difficult to convey quite how much my arrival at the site mattered to me.“ Es seien Cousteaus technische Erfindungen und sein PR-Talent gewesen, „that made the excavation a test case on the history of submarine archaeology.“ Bereits bei ihrem ersten Tauchgang fand sie Teile des Schiffsrumpfs am Meeresgrund, die während der Kampagne nicht geborgen worden waren. Cousteau sei ein „many sided genius“ und als Taucherin sei sie ihm vieles schuldig, dennoch übte sie auch Kritik: „This said, it would be preferable to pass over his remarks on archaeology.“ Cousteaus Beiträge im *National Geographic* hätten Anfang 1960 noch die besten Informationen über die Kampagne veröffentlicht, wenngleich „they almost convince readers that submarine excavation is impractical.“ Sie gestand der Grabung zu, dass es sich dabei um „pioneer work“ gehandelt habe, aus archäologischer Sicht seien die Ergebnisse jedoch vollkommen unzureichend. Vor allem befürchtete sie, dass die Unterwasserarchäologie durch Inszenierungen wie die von Cousteau, an der Dumas teilnahm, die Möglichkeit verspielte, wissenschaftlich ernst genommen zu werden. Beispielsweise wisse man im Nachhinein – und damit widersprach sie Benoit – dass die *sucaseuse* nicht analog zur terrestrischen Grabung verwendet werden dürfe. Am Meeresboden lassen sich etwa keine Gräben und Schächte anlegen, da man dort keine stabilen Seitenwände erzeugen kann. Folglich kommt es zu Rutschungen, die den gesamten Grabungszusammenhang durcheinanderbringen. Doch nicht solche unausweichlichen Fehler in der Pionierarbeit seien unentschuldbar, sondern die mangelhafte Sorgfalt bei der Durchführung der Kampagne und ihrer Dokumentation: „Questions will arrive, which could have been answered if excavation had been systematic, and negative results will reflect on underwater archaeology in general.“ (Frost 1963: 232–239).

Benoit sprach in seiner 1961 veröffentlichen Publikation *Fouilles sous-marines. L’épave du Grand Congloué à Marseille *über die Unterwassergrabung von „le mauvais état de conservation de l’épave“, die weder die Rekonstruktion des Rumpfs noch des Schiffsprofils erlaube. Das bei Cousteaus TV-Liveübertragung gehobene Rumpfstück des Wracks von 1,60 Meter Länge sei ein Einzelstück, das keine weiteren Schlussfolgerungen zuließe, weil Kontexte fehlten. So sei es etwa nicht möglich, die Fragen zu klären, ob und wenn ja, in welcher Form eine Längsverstärkung des Rumpfes vorlag, wie sie von Handelsschiffen bereits bekannt war (Benoit 1961: 151–154). Benoit sah sich nur in der Lage, die Holzarten des Fundes zu identifizieren (Eiche, Aleppo-Kiefer und Akazie) und wenige Konstruktionsdetails mit dem Forschungsstand zum antiken Schiffsbau in Verbindung zu setzen. Dieser Forschungsstand umfasste – neben einigen gut dokumentierten antiken Wracks wie jenen der Nemi-Schiffe – vor allem eine „Fülle an epigrafischem Vokabular und realen wie figurativen Darstellungen des ägyptischen Nautismus“ sowie Textstellen verschiedener antiker Autoren. Obwohl letztlich viele Fragen zum Schiffsbau ungeklärt blieben, sieht Benoit in der „architecture navale“ den „l’apport le plus nouveau“ der Kampagne bei Grand Congloué. Die Schiffsarchäologie sei „une science toute nouvelle.“ (Benoit 1961: 199).

Frost tauchte Anfang 1960 – teilweise in Dumas’ Begleitung – mehrmals zu dem Wrack hinab. Dort stellte sie schnell fest, dass große Stücke der mit Blei beschlagenen Holzteile des antiken Wracks noch in den oberen Schichten des Meeresbodens lagen und damit leicht zugänglich waren. In nur wenigen Tauchgängen konnte sie Teile der Konstruktion detailliert vermessen und Proben nehmen. Ihr Urteil über die Arbeit am Grand Congloué war infolgedessen ausgesprochen kritisch. Es seien viele Hinweise ignoriert worden. Wenn die Ausgrabung des Wracks systematisch erfolgt und alle Verhältnisse in situ aufgenommen worden wären, wäre es möglich gewesen, präzise Ideen von der Form des Schiffes zu entwickeln: „The answers are there, buried in the sand. The point at issue is: will normal methods of archaeological recording give results underwater as they do on land? I think the answer is ‚yes.‘“ (Frost 1963: 252) Auch kritisierte sie Benoits mangelnde Sorgfalt sowohl bei der Arbeit unter Wasser sowie bei der Dokumentation: „Judging from what he has seen on land, one can sympathize with his pessimistic conclusion, but it would be premature to accept capitulation before at least trying to apply the recording routine of modern field archaeology.“ (Frost 1963: 238).

Ende des Jahres 1959 waren die ersten Schritte für den Versuch einer systematischen unterwasserarchäologischen Kampagne am Kap Gelidonya gemacht. 1958 und 1959 hatten Frost und Throckmorton die gründliche Erkundung der Fundstellen besorgt. Frost hatte Untersuchungen von Bronzefunden in einem Oxforder Institut organisiert und sich anhand des Wracks von Grand Congloué noch einmal versichert, dass ein systematisches Vorgehen neue Erkenntnisse bringen würde. Zudem war mit Dumas einer der bekanntesten Taucher der Welt gewonnen worden. Aber ohne ausreichende finanzielle Mittel würden sie ihr Ziel einer methodisch sauber durchgeführten Grabung nicht umsetzen können.

## Start der Kampagne 1960

Über den Jahreswechsel planten Frost und Dumas in Sanary-sur-mer den Material- und Zeitbedarf der Kampagne und notierten den Forschungsstand. Das Ergebnis war ein Forschungsantrag, der „the present shortcomings of method in scientific underwater research“ beklagte und den Throckmorton dem Museum der University of Pennsylvania vorlegte. Unter Archäolog*innen, aber auch Tauchenthusiast*innen herrsche die Überzeugung, „that merely to loot an ancient wreck for its cargo is completely destructive.“ Neue Techniken der Unterwasserfotografie und des Zeichnens unter Wassers seien verfügbar und könnten weiterentwickelt werden. „It is now possible to make fairly accurate measured drawings under water and an archaeological expedition at work on one of these wrecks would have in mind to work very slowly, recording all details by drawing the structure of the ancient ships about which we know nothing.“ Das Heben antiker Schiffe sei unmöglich; sie seien dafür zu fragil. Deshalb sei das Ziel, in situ detaillierte Zeichnungen anzufertigen und so Fundorte gut zu dokumentieren, „instead of merely destroying the wrecks of ancient ships.“ Insgesamt hätten die Vorarbeiten zur Identifizierung von 38 Wracks geführt: „The earliest wreck, of the Bronze Age, is unfortunately at the moment quite unique.“ Proben der Bronzen legten nahe, dass es sich um einen zypriotischen Rohstofftransport aus dem zweiten vorchristlichen Jahrtausend handelte. Jetzt dränge die Zeit: Zwei ähnliche Wracks „were dynamited by the sponge-fishers for the sake of the salvage-value of the cargo […]. The opportunity is unique and important and one which must be acted upon with all speed.“^11^

Im Februar 1960 schrieb Throckmorton, dass die University of Pennsylvania ihm 20.000 US-Dollar zugesichert habe. Er, Frost und Dumas erhielten zudem einen neuen Grabungsleiter: George Bass hatte gerade seinen Master in Archäologie absolviert und begann mit der Arbeit an einer Dissertation.^12^ (Bass 1967b: 118) Frost konnte zudem die erfahrene Archäologin Joan du Plat Taylor (1906–1983) des University College London gewinnen. Mit US-amerikanischem Geld, den studierten Archäolog*innen Bass und du Plat Taylor, der Unterstützung seitens ihrer Kontakte aus Izmir – namentlich dem Tauchclub und dem Direktor des archäologischen Museums – waren die Voraussetzung für eine offizielle türkische Grabungsgenehmigung erfüllt. Tauchausrüstung wurde angekauft und in die Türkei gebracht, wo erneut Kapitän Kemal und seine Crew angeheuert wurden. Am 4. Juni 1960 legte das Team schießlich los (Bass & Throckmorton 1967: 21). Zwei Schiffswracks wurden genauer unter die Lupe genommen, darunter das hier im Mittelpunkt stehende bronzezeitliche Wrack, das den Spitznamen „Bronze Wreck“ erhielt.

Die Lebens- und Arbeitsbedingungen waren beschwerlich. Die Ausgrabenden schlugen ihr Lager auf einem unbewohnten Strand unter einer Klippe in der Nähe der Fundorte auf. Der schmale Sandstreifen blieb im Sommer für gewöhnlich trocken, doch zeitweise drückte ein außergewöhnlich starker Wind Meerwasser ans Ufer, welches das Camp überschwemmte. Größere Schäden an Zelten, Material oder gehobenen Funden waren nicht zu verzeichnen, den Alltag erschwerten solche Fluten dennoch. Und nicht nur die Natur, auch die Technik bereitete Schwierigkeiten. Ein großer Kompressor steckte lange im Zoll fest, vorhandene Kompressoren waren regelmäßig defekt, sodass selten alle Flaschen mit Druckluft befüllt werden konnten, was Tauchgänge verkürzte oder ausfallen ließ (Bass 1961: 7; Bass & Throckmorton 1967: 6–7). Gleichzeitig stand die Mannschaft unter einem enormen Zeitdruck, weil sie befürchtete, dass alles am Meeresboden Zurückgelassene geplündert werden würde, sobald man abrückte.

Das „Bronze Wreck“ lag in einer Tiefe von 26 bis 28 Metern. Als erfahrenste/r Taucher*in plante Dumas die Tauchgänge und schulte das Team. Er entschied, dass zwei Tauchgänge pro Tag möglich seien. Der erste dauerte maximal vierzig Minuten, wobei hiervon der Abstieg samt sechs Minuten Dekompression in drei Metern Tiefe abzuziehen sind, sodass für rund dreißig Minuten gearbeitet werden konnte. Erst nach einer mindestens sechsstündigen Pause erlaubte er einen zweiten, verkürzten Tauchgang (Bass & Throckmorton 1967: 22). Der Grund für die kurzen Arbeits- und langen Pausenzeiten lag in der Wirkung des Tiefendrucks auf den Körper. Beim Auftauchen gast Stickstoff, der sich in der Tiefe im Blut löst, wieder aus, wenn der Druck auf dem Weg zur Oberfläche abnimmt. Typische Folgen dieser sogenannten Dekompressions- oder Caissonkrankheit sind heftige Gelenk- und Kopfschmerzen. Seit dem ausgehenden 19. Jahrhundert beschrieben Physiologen dieses Phänomen (von Lünen 2010: 31–81). In der Folge suchten Industrie- und Marinetaucher bereits vor dem Ersten Weltkrieg routinemäßig der Taucherkrankheit vorzubeugen, indem sie während des Aufstiegs Pausen einlegten, damit die Gaskonzentration im Blut sich wieder dem Umgebungsdruck angleichen konnte (Möser 1992: 202–203). Die von Mediziner*innen entworfenen und beständig verbesserten Tabellen, welche Aufstiegsgeschwindigkeiten vorgaben, waren auch mehrere Jahre nach dem Zweiten Weltkrieg bei griechischen und türkischen Schwammtauchern noch unbekannt. Cousteau und Dumas schilderten in ihren populären Darstellungen des Meeres die deutlich erkennbaren körperlichen Folgen der Taucherkrankheit: „Wenn sie dann aus ihren imposanten Taucheranzügen ausstiegen, waren sie verrenkte kleine Männer, die von der Caisson-Krankheit befallen waren.“ (Cousteau & Dumas 1953: 47).

Doch nicht allein der Wechsel zwischen Wasser und Atmosphäre birgt Gefahren. Strikte Disziplin muss beim Tauchen auch deshalb eingehalten werden, weil der Tiefendruck die Sinne stört. Bei der Untersuchung des Wracks bei Grand Congloué war der italienische Marinetaucher Jean-Pierre Servanti gestorben, als er einen losgerissenen Anker zu heben versuchte (Cousteau 1954: 17–18). Ursache solcher Unfälle war oft der Tiefenrausch – eine Stickstoff- oder Sauerstoffvergiftung, deren frühe Untersuchung und Beschreibung unter anderem der US-amerikanische Marinearzt Albert R. Behnke geleistet hatte. Cousteau und Dumas notierten über den Tiefenrausch, er sei wie ein Alkoholrausch ohne Kater: „[W]enn man der gefährlichen Zone erst einmal entronnen ist, wird man sogleich klar im Kopf“. Die Gefahr des Tiefenrausches bestehe darin, dass der/die Taucher*in die lebensfeindliche Umgebung vergisst und beispielsweise den Atemschlauch aus dem Mund nimmt, um sich der „ivresse des grandes profondeurs“ „großmütig anzubieten.“ Körperliche Verzückung und Lebensgefahr sind in der extremen Umwelt der Tiefe miteinander verwoben: „Ich liebe [den Tiefenrausch] und fürchte ihn zugleich wie das schlimmste Verhängnis. Er zerstört den Lebensinstinkt.“ (Cousteau & Dumas 1953: 42) Auch diese Beeinträchtigungen der Sinne waren Rahmenbedingungen der archäologischen Arbeit unter Wasser und hatten direkten Einfluss auf die Forschungspraktiken.

## Gegenstände und Techniken der archäologischen Forschung in der extremen Unterwasserumwelt

Als die Taucher*innen am Kap Gelidonya mit der Untersuchung des Wracks begannen, orientierten sie sich an den Forschungszielen der Archäologie. Heute, so der Ägyptologe und Kulturwissenschaftler Jan Assmann, sei der Grabungskontext stets „der wichtigste Schlüssel zur Bestimmung“ der Artefakte (Assmann 2015: 101). Dieses Paradigma der Feldarchäologie hat sich seit den 1890er-Jahren mit der Durchsetzung stratigrafischer Methoden zunehmend behauptet (Gran-Aymerich 2007: 473). Mit dieser Form der archäologischen Forschung stiegen auch die Anforderungen an die Dokumentation. Kathleen Kenyon, bei der Frost das Handwerk der archäologischen Feldarbeit gelernt hatte, notiert in ihrem 1952 erstmals veröffentlichten Lehrbuch: „All excavation is destruction. […] [T]he evidence is destroyed altogether unless it has been properly observed, recorded and subsequently made public.“ (Kenyon 1961: 68) Das Ziel einer Grabung sei, „the relation of finds“ zu bestimmen und zu dokumentieren (ebd.: 115). Diese Verhältnisse der Funde zueinander wurden zum „epistemischen Ding“ – also zum „Gegenstand der Forschung im engeren Sinne“ (Rheinberger 2006: 27), nach dem auch vor der türkischen Küste beim Hinabsteigen in die Tiefe gesucht wurde.

Für Honor Frost wurde die visuelle Repräsentation der Fundstätte zum zentralen Thema. Sie folgt damit Kenyon, die bereits in ihrem Lehrbuch Karten und Pläne zum Gegenstand des Erkenntnisinteresses erklärt hatte. Ziel einer Grabung sei „to put the site on the map, to make a plan of the site and its structures, and to show the area excavated.“ (Kenyon 1961: 115) Ihre Schülerin Frost bezeichnete sich wiederholt als *draughtsman*, was die Bedeutung unterstreicht, die sie der Herstellung gezeichneter Repräsentationen zuwies. Zumal war sie über ihr praktisches Zeichentalent erst zur archäologischen Feldarbeit gekommen. Kenyon betont, dass die Fähigkeit zur Aufnahme und Dokumentation von Fundorten ein Weg sei, die Archäologie zum Beruf zu machen: „If, for instance, someone who wished to make archaeology his career were also a good draughtsman, he could easily tide over an interval before obtaining an appointment.“ (Kenyon 1961: 59).

**Figure 2:**
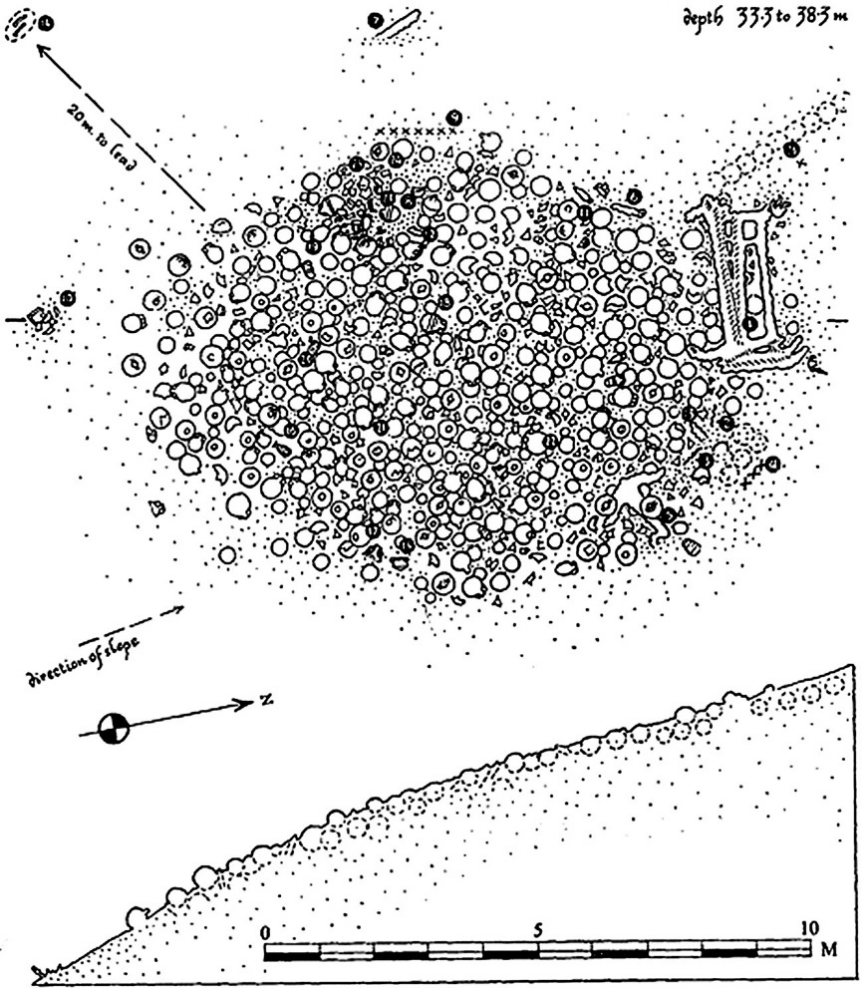

Am 3. Januar 1961, wenige Monate nach der Rückkehr aus der Türkei, sprachen Frost, Dumas, du Plat Taylor und Kenyon gemeinsam im Radioprogramm der BBC über die Unterwasserarchäologie. Die besondere Bedeutung von Karten unterstrich wiederum Kenyon. Sie lobte Frosts „beautiful plans“ und fragte, wie sie diese hergestellt habe. Frost antwortete, dass Kenyon selbst den Anstoß dazu gegeben hatte. Es seien die Planzeichnungen in Jericho gewesen, „that sent me to Turkey to see whether I could do the same thing on wrecks in deep water.“^13^ Kenyons Anerkennung als „schön“ unterstreicht noch einmal die Bedeutung der Pläne als epistemische Dinge (vgl. Abb. 2).

Von den Karten ausgehend kamen die Diskutant*innen der BBC-Runde auch auf die Rolle der Fotografie zu sprechen. Frost bemerkte, dass es zwar tolle journalistische Unterwasserfotos gäbe, aber keine guten Aufnahmen von vollständigen Wracks. Bei den nötigen großen Abständen könne die Kamera unter Wasser auch bei idealen Bedingungen keine Details mehr einfangen. Auch aus mehreren Aufnahmen zusammengesetzte Bilder scheiterten daran, das Wichtige vom Unwichtigen zu unterscheiden. In der Regel machten schlechte Sicht und das Fehlen von Schatten es nahezu unmöglich, auf den Fotos wichtige Details zu erkennen. Nützlich für die Feldarbeit seien einzig Fotos von ausgewählten kleinen Ausschnitten: „What the draughtsman needs are photos as aide memoires.“^14^ Damit mit diesen Fotos etwas anzufangen ist, müssten sie nach den Angaben des/der Zeichners/Zeichner*in erstellt und am gleichen Tag entwickelt werden. Dann ließen sich die unter Wasser angefertigten Skizzen nach dem Auftauchen durch Fotos ergänzen und verbessern. Frost spricht damit ein bekanntes Problem der Wissenschaftsgeschichte an, nämlich den unklaren epistemischen Status der Fotografie (Brusius 2015). Sie teilte die Annahme, dass Fotos keine Interpretationen lieferten, jedoch als Deutung zu verstehen seien, welche die Wissenschaft braucht:

The underwater draughtsman’s job is to select and measure the significant within his limited time; this the camera cannot do. The photographer’s job is to take [photos of the] same area at [the] same stage of excavation, so that [the] draughtsman can enter the details he has not had time to draw.^15^

**Figure 3:**
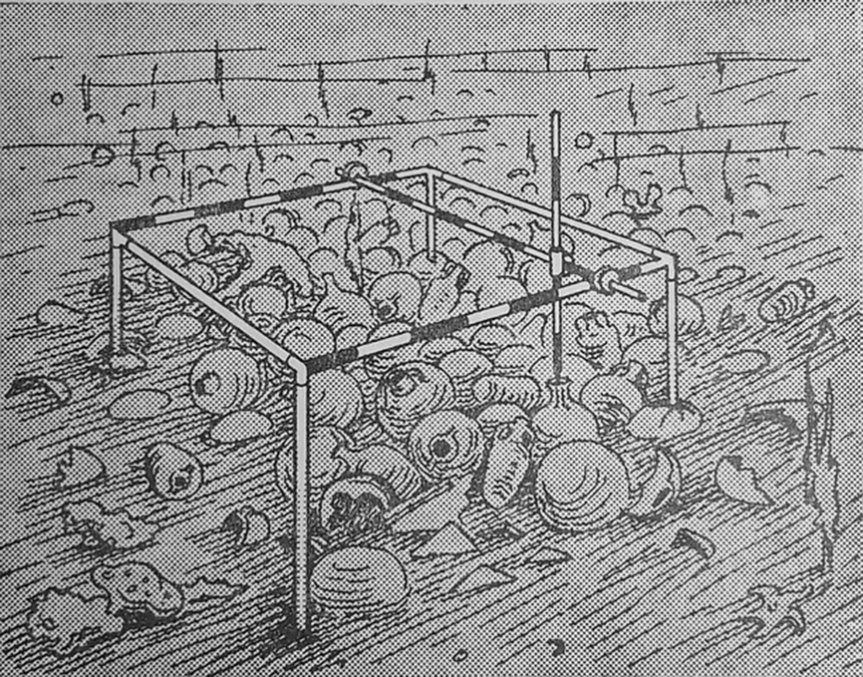

Zur Herstellung von Karten, die die spezifischen Gegebenheiten des Fundorts interpretierten und repräsentierten, war Technik notwendig. Neben der Fotografie war das die Tauchtechnik. Hans-Jörg Rheinbergers Beobachtungen zu den Laborwissenschaften helfen, die Verhältnisse von Forschungsgegenständen und Forschungstechniken besser zu begreifen. Auch im Fall der Feldforschung gilt, dass „technische […] Bedingungen […] die Wissensobjekte in doppelter Hinsicht [determinieren]: Sie […] lassen sie so erst als solche hervortreten, sie begrenzen sie aber auch und schränken sie ein.“ (Rheinberger 2006: 29) Die antiken Wracks und ihre Ladung wurden erst untersuchbar, als Tauchtechnik den Zugang zur lebensgefährlichen Unterwasserumwelt gestattete. In ihrer Anwendung von Technik waren die Taucher*innen am Kap Gelidonya „‚Bastler‘, Bricoleure, weniger Ingenieure.“ (Rheinberger 2006: 34) Sie passten ihre Hilfsmittel beständig an die Forschungsfragen und die natürliche Umgebung der Fundstelle an. Frost ging aufgrund dieser Erfahrung davon aus, dass jedes Wrack neue technische Lösungen nötig macht: „There is no universal panacea for gadgets.“^16^ Dumas und sie hatten beispielsweise im Vorfeld ein Raster für die Vermessung von Fundstellen entwickelt, das letztlich nicht anwendbar war. Das „Bronze Wreck“ lag auf einem steinigen Untergrund, weil sie in Sanary-Sur-Mer jedoch lediglich ein Verfahren zur Fundaufnahme im schlammigen Meeresboden entwickelt hatten, mussten sie nun vor Ort ein neues System erdenken und erproben. Ihre Versuche resultierten in der Herstellung eines Metallrahmens, der mit Wasserwaagen versehen war und dank verstellbarer Beine auch auf unebenem Untergrund waagerecht aufgestellt werden konnte. Mit einem Lot ließen sich zudem relative Höhenangaben nehmen (Abb. 3).

Die unterschiedliche Beschaffenheit des unterseeischen Bodens machte vielfach Anpassungen der Grabungstechnik notwendig. Die *sucaseuse* der Ausgrabung am Grand Congloué ist ein weiteres Beispiel dafür, wie vorhandene Technik umgebaut und angepasst wurde, damit die Untersuchungsgegenstände überhaupt erst aus den Ablagerungen geholt werden konnten. Das grundlegende Prinzip, mit Unterdruck Schlamm vom Boden an die Oberfläche zu befördern, war etwa in Form von Spülbaggern schon lange bekannt, aber diese Konstruktion wurde angeglichen. Taucher*innen konnten den Schlauch präzise führen und den Saugstrom kontrollieren; die obere Öffnung wurde um Netze ergänzt, die hochgewirbelte Fundstücke auffingen. Ein solcher Airlift wurde auch beim „Bronze Werck“ benutzt. Hier fand er – wie oben bereits angesprochen – nicht für die Herstellung von Schnitten Verwendung, sondern zur schichtweisen Räumung der Fundstelle. Auch einzelne Arbeitsschritte wurden an die neue Umwelt angepasst. Frost und ihre Mitstreiter*innen wichen damit von Kenyons zentralen Ideen ab, wie Grabungen durchzuführen seien. Um die Siedlungsschichten einer Grabungsstätte zu identifizieren, trieb Kenyon zunächst schmale Gräben bis auf die Basis der Fundstelle. Ausgehend vom genau dokumentierten Schnitt wurde dann die Arbeit fortgesetzt (Dever 2004: 540). Eine Anpassung dieser Vorgehensweise wurde unter Wasser auch deshalb nötig, weil es bei einem Wrack logischerweise keine Siedlungsschichten gibt. Chronologien, wie sie von der Landarchäologie seit dem Ende des 19. Jahrhunderts regelmäßig hergestellt wurden, spielten deshalb für den vorliegenden Untersuchungsfall keine Rolle (vgl. Gran-Aymerich 2007: 471–472).

Eine Technik wie die *sucaseuse *besteht zudem nicht allein aus technischen Apparaturen, sondern verdankt ihren Erfolg immer auch der gekonnten Anwendung. Frost notiert, dass der Einsatz des Geräts theoretisch einfach, in der extremen Umwelt unter Wasser praktisch jedoch sehr schwierig ist. Bereits geringe Veränderungen der Haltung oder der Einsatztiefe verändern die Funktionsfähigkeit des Airlifts:

The machine does or does not work, according to the angle at which it is rigged in the water […]. Further, the force is affected by depth, as air expands at different rates at different water pressures, so that the machine’s performance varies from one part of a wreck to another. Taken together, these factors make an air-lift beyond simple mechanical control. (Frost 1963: 21)

Die naturräumlichen Eigenschaften unter Wasser unterschieden die Feldforschung von „gewöhnlicher“ Archäologie am Festland: „[W]e must recognize the fundamental difference between the practice of excavation and the kind of [underwater] research mentioned.“ Zunächst gebe es Gemeinsamkeiten: Hier wie dort müssten manchmal Hunderte Tonnen Sand, Stein und – im Fall von Wracks – Ladung bewegt werden. Auch müsse in beiden Fällen die Stelle vor Beginn der Arbeiten grob vermessen werden. Allerdings verhindern schwierige Sichtverhältnisse und das Fehlen des Horizonts unter Wasser die Anwendung von Theodoliten. Stattdessen müsste die Fundstelle von Fixpunkten aus mit Maßbändern abgeschwommen werden.^17^ Bei der Unterwasserarchäologie braucht es insgesamt deutlich mehr Technik und Menschen, die sie bedienen können: „Not only a sizeable boat and equipment are needed for this heavy labour but also skilled seamen and divers practised in the handling of tools.“ (Frost 1963: 16).

Während das technische Equipment die Untersuchungsgegenstände erst zutage treten ließ, indem Fundstätten etwa durch Tauchtechnik zugänglich gemacht, mit der *sucaseuse* vom Schlamm befreit und mit neuen Hilfsmitteln vermessen wurden, so beschränkte es andererseits die Forschungspraktiken und damit auch die zu findenden Objekte. Das Gerätetauchen konnte mit der 1960 vorhandenen Ausrüstung nur bis zu einer Tiefe von rund 40 Meter sicher durchgeführt werden. Alle Wracks, die tiefer lagen, schieden damit als Untersuchungsobjekte aus. Diese Grenze wurde in den folgenden Jahrzehnten weiter ausgedehnt, besteht aber ganz grundsätzlich immer noch. Auch setzen die von Frost und Dumas entwickelten Techniken auf die visuelle Fundaufnahme. Mit Sonar und anderen Praktiken ist es heute möglich, selbst Funde zu orten und zu kartieren, die dem Auge verborgen sind. Nicht so 1960 an der türkischen Küste: Während Dumas die schichtweise Räumung des Fundes anleitete und du Plat Taylor die Verzeichnung der Funde auf dem Trockenen übernahm, war es vor allem Frost, die mit einfachen Hilfsmitteln die Kartografierung der Fundstelle besorgte.^18^ Sie notierte in diesem Zusammenhang die Einschränkungen ihrer Arbeit: „Apart from explaining the site, the main value of my work was again, that it taught me the exact limitations inherent in deep-water recording.“ (Frost 1963: 174).

Die etablierten Techniken der Vermessung von Fundstätten konnten nicht einfach auf die Unterwasserwelt übertragen werden. Zudem war Frost der Meinung, dass Know-how und technische Improvisation gerade angesichts der spezifischen naturräumlichen Umstände allein nicht ausreichten. Es gäbe „one last important point of principal: working underwater, every measurement has to be re-checked.“ Drei Eigenschaften von Technik und Umwelt seien es, welche die Praktiken der Unterwasserarchäologie beschränkten: „disorientation due to lack of horizon, distorted vision, and the effect of depth on the mind.“ (Frost 1963: 174).

Erstens würde die Orientierung dadurch erschwert, dass der Taucher sich in alle drei Richtungen bewegt: „His disorientation can be such that, in deep water or poor visibility, he may not even know whether he is moving up or down. […] Lack of horizon removes the very basis of survey techniques.“ (Frost 1963: 174–175) Frost notiert, dass sie Entfernungen von sechs bis zwölf Meter mit einem Maßband gemessen habe. Man könne jedoch nicht sicher sein, dass die Maßbänder wirklich horizontal liegen. Abfallendes oder aufsteigendes Gelände sei fast nicht zu erkennen, wenn man darüber hinwegschwimmt, „so there was bound to be a discrepancy between the overall measurements and the sum of the detailed drawings made on the sea bed.“ Diese Messfehler müsse man nach der Aufnahme der Messungen sorgfältig ausgleichen. Hinzu kam, dass die Tiefenbestimmung nicht genau möglich war. Diese Probleme verstärkten sich, wenn man begann, Schicht für Schicht die Ladung abzutragen: „On all wrecks the main difficulty is this measurement in depth. It becomes more pronounced when layer after layer of cargo is lifted and each has to be related to the last both in plan and in section.“ (Frost 1963: 183).

Die zweite Schwierigkeit, mit der umzugehen man lernen müsse, seien visuelle Verzerrungen und Uneindeutigkeiten. Die Maske vergrößert alle Dinge um ein Viertel, während sie den Sichtwinkel einschränkt. Die Folge für den/die tauchende/n Archäologen*in beschreibt Frost bildlich: „[H]e becomes like a horse wearing blinkers.“ Zudem gibt es unter Wasser keinen Schattenwurf, sodass das Relief von Oberflächen nahezu unsichtbar ist. Die verwendeten Techniken beschränken auch hier die Forschungspraktiken: „[T]he effect it has on a draughtsman trying to plot only a few square metres is serious.“ (Frost 1963: 175).

Drittens setzt beim Abtauchen relativ früh eine mentale Beeinträchtigung ein. Je tiefer man in die fremde Umwelt hinabtaucht, desto mehr würden die Sinne gestört: „[R]eactions become automatic once depth affects the mind.“ Hiergegen könne man sich am einfachsten rüsten, indem man alles im Voraus plant. „I write objectives like a shopping list on my tablet or flippers and cross them off as they are accomplished.“ (Frost 1963: 176) Frost erwähnt, dass Routinen halfen, diese vielfältig prekären Konditionen unter Kontrolle zu halten. Unter Wasser nutzte sie eine selbst erdachte Behelfstechnik, indem sie mit einem Filzstift auf einem durchsichtigen Plastikblatt zeichnete, das wiederum auf ein Metallbrett geklemmt war. Nach dem Auftauchen machte sie sich sofort daran, ihre neusten Skizzen in die vorhandenen Pläne einzufügen. Der erste Tauchgang des Tages begann wiederum mit der Überprüfung und Verbesserung dieser Pläne.^19^

Die Wissenschaftsgeschichte betont in der Untersuchung wissenschaftlicher Praktiken seit Langem, dass technische und epistemische Dinge in Austauschbeziehungen und im wechselseitigen Übergang zueinander stehen. Bruno Latour prägte unter anderem zur Kennzeichnung verschwommener Grenzen zwischen Aspekten der Technik, Natur oder Gesellschaft den Begriff des Hybriden (vgl. etwa Latour 1995: 7–9) und auch Rheinberger notiert, dass seine Unterscheidung lediglich „funktional“ zu verstehen sei (Rheinberger 2006: 31). Die Beziehungen gestalten sich bei der Forschung in der extremen Umwelt unter der Meeresoberfläche ähnlich. Hier sind jedoch neben archäologischen Artefakten und Tauchtechniken auch der Naturraum und der/die Taucher*in so miteinander verschränkt, dass sie als Elemente einer wechselseitigen Abhängigkeit verstanden werden können. Diese Verwobenheit illustriert abschließend noch einmal Frosts Beschreibung des Umgangs mit der *sucaseuse*: „A diver puts himself against a force of nature.“ Die Arbeit sei auf wenige Minuten konzentriert, in denen der/die Taucher*in „knowns that he dare not get out of breath. Digging his ‚big hole‘ with an air lift, he has to hold his powerful machine“. Dabei hat man den freizulegenden Gegenstand ständig vor Augen. So versuche man etwa, während eines Tauchgangs eine Amphore aus dem Schlamm zu befreien. Bei solch konzentrierter Arbeit geht leicht das Bewusstsein für die extreme Umwelt verloren, in der man arbeitet: „He pulls, until his objective becomes an obsession; judgement, already impaired by depth, hardly governs his action. If he is experienced he will realize what is happening to him; if not, being alone on the bottom, he may wrench the amphora and break it.“ Oder, schlimmer, man bringt sich selbst in Gefahr, denn ein/e Unterwasserarchäologe/*in „becomes emotionally part of his big hole.“ (Frost 1963: 259).

## Disziplinengenese, US-amerikanische Hegemonie und begrabene Kriegsbeile

Nach Abschluss der Kampagne im Spätsommer 1960 sahen sich Frost, Dumas und du Plat Taylor vom neuen US-amerikanischen Grabungsleiter George Bass an die Seite gedrängt. Am 14. März 1961 schrieb Dumas an Frost, er habe Bass’ frisch veröffentlichte erste Publikation von der Unterwassergrabung in die Hände bekommen und war ausgesprochen unzufrieden: „He speaks always of ‚we‘ and hardly mentions your name or mine, we all are on the same level, which let the reader think he was a great man and did everything.“^20^ Am 17. März setzte er noch einmal nach, was unterstreicht, wie verärgert er war: „You did all drawings and maps, I did the entire excavation, if not discovery […]. George is not a fool, he will follow the idea and pretend he invented and developed it […].“^21^ Und in der Tat werden bei der Lektüre von Bass’ Schriften die Leistungen der Initiator*innen der Grabungskampagne fast nicht ersichtlich (Bass 1967a).

Dumas und Frost kooperierten nie wieder mit Bass und kehrten auch nicht wieder zu den Wracks vor Bodrum zurück. Frost kümmerte sich in London um den Nachlass ihres verstorbenen Vormunds und initiierte neue Unterwassergrabungen und Kooperationen. Bis an ihr Lebensende war sie in der Community aktiv (vgl. Blue 2019). Mit Throckmorton kam es zwar nicht zum Bruch, aber die Spannungen zwischen ihnen waren enorm. Im November 1960 schrieb Throckmorton aus Mykonos: „As far as our personal dealings are concerned, the thing to remember is that we DID something, for the first time ever, and that it was for all its defects, probably really good. […] Underwater Archaeology is bigger than any of us.“^22^

1969 brachten die beiden mit weiteren Co-Autoren schließlich noch einen Band zur Unterwasserarchäologie heraus (Frost & Throckmorton 1969). In Bodrum erinnert das unterwasserarchäologische Museum in der Kreuzfahrerfestung heute an Throckmorton als jenen, der die Vision für das Museum hatte.^23^ Throckmorton arbeitete seinerseits wiederholt mit Bass zusammen. Beide waren neben anderen Gründungsmitglieder des Hellenic Institute of Maritime Archaeology und tauchten auch mit Arthur C. Clarke vor Sri Lanka nach Wracks (Norton 1999: 260–264). Bass wurde in den Folgejahren zu einem der einflussreichsten Unterwasserarchäologen. 1966 veröffentlichte er die populär gehaltene Einführung *Archaeology Under Water*, die bereits im gleichen Jahr in deutscher Übersetzung vorlag (Bass 1966). 1972 gründete er an der Texas A&M University das Institute of Nautical Archaeology, das neben einer Vielzahl von Grabungskampagnen auch intensiv die Entwicklung neuer Techniken vorantrieb.

Dumas veröffentlichte sein Handbuch der Unterwasserarchäologie 1964, nach meinem Wissen das erste Werk dieser Art (Dumas 1964). Er setzte sich zudem für die Institutionalisierung der Unterwasserarchäologie ein, etwa indem er eine Sektion der World Underwater Federation mitbegründete. Er kehrte der Archäologie unter Wasser dennoch bald den Rücken zu und fuhr wieder mit Cousteaus Equipe um die Welt. Frustriert, dass seine Leistungen von der Fachwelt ignoriert wurden, schrieb Dumas bereits am 20. Juni 1961 an Frost: „If we were living fifty years ago I should say I am the man of the underwater archaeology and do the job“. Es bleibe ihm nichts anderes übrig, als jungen Archäolog*innen das Tauchen beizubringen, „[to] give them lectures on underwater archaeology[,] show them typical sites and lead the job as long as they are incompetent.“^24^

Dumas’ Beispiel zeigt exemplarisch eine Folge der Disziplinengenese in den Wissenschaften (vgl. Fehr & Orland 2010). Die Untersuchung des „Bronze Wreck“ markiert einen Moment in der Entstehung des neuen Forschungsbereichs Unterwasserarchäologie, der durch die Übernahme etablierter Forschungsmethoden „und ihre[r] Einbettung in neue Forschungszusammenhänge“ (Reinhard 2011: 127) gekennzeichnet war. Diese Zusammenhänge waren durch die Eigenschaften der extremen Unterwasserumwelt einerseits sowie die Überlebens- und Forschungstechniken andererseits gekennzeichnet. Dumas war nicht nur ein erfahrener Taucher; er war auch eine/r der frühen Akteur*innen, die eine Systematisierung der archäologischen Feldforschung unter Wasser vorantrieben, wie sein 1964 veröffentlichtes Handbuch der Unterwasserarchäologie zeigt. Dennoch erhielt er in der akademisch verankerten Archäologie keine fachliche Anerkennung. Zwar verfügte er über das praktische Wissen, aber ihm fehlten Universitätsdiplome und akademische Netzwerke. Bei Frost war die Situation weniger eindeutig; sie übernahm keinen Lehrstuhl, wurde aber als „Quereinsteigerin“ eine aktive und gut vernetzte Unterwasserarchäologin – und das aus zwei Gründen: Sie war finanziell unabhängig, zudem war Mitte des 20. Jahrhunderts das britische Wissenschaftssystem wohl auch offener als das auf dem Kontinent. Frosts Lehrerin Kenyon, deren Ausgrabungen in Palästina in der Nachkriegszeit zur Schule einer ganzen Generation von Archäolog*innen wurden, betonte den Vorrang, den praktische Grabungsarbeit vor theoretischem Wissen und Sprachkenntnissen hatte (Dever 2004: 532).

Wie eingangs bemerkt, kann der vorliegende Aufsatz nur erste Einblicke in die Formierungsphase der Unterwasserarchäologie als Disziplin liefern. Auf weitere Desiderata weist etwa der Eintrag „Maritime Archaeology“ in der *Encyclopedia of Global Archaeology *hin. Hier heißt es, dass „[t]he emergence of maritime, underwater or nautical archaeology as a field or subdiscipline within archaeology“ mit George Bass’ Arbeit im Mittelmeer verknüpft sei. „His research project was the first underwater excavation of a shipwreck directed by a diving archaeologist.“ (McKinnon 2014: 415) Die Kampagne am Kap Gelidonya zeigt, dass sich bei einem genaueren Blick hinter solchen Generalisierungen historische Ereignisse verbergen, die mehr sind als reine Helden- und Pioniergeschichten. Neben den hier gewählten Schwerpunkten – das heißt dem Fokus auf einer Kampagne und den Praktiken der Feldforschung – fehlen auch historische Arbeiten, die biografie- oder institutionengeschichtliche Aspekte der Unterwasserarchäologie untersuchen. Letztlich sind solche Arbeiten für belastbare Aussagen zur Geschichte dieser Forschungsdisziplin als Ganzes notwendig.

Typisch für die Unterwasserarchäologie der Zeit um 1960 scheint mir zu sein, dass Tauchenthusiasmus und akademisches Interesse gleichermaßen eine Voraussetzung waren. Dass die persönliche Begeisterung für die Unterwasserwelt oftmals inhärenter Teil dieser Disziplin ist, lässt sich bis heute anhand zahlreicher wissenschaftlicher Biografien beobachten. Frost sprach sogar davon, dass „some form of diving became a necessary part of my life. In the circumstance archaeology had its uses for me.“ (Frost 1963: 29) Diese Bedeutung des Umweltraums und seines direkten Erlebens für Forscher*innen fügt sich in die Beobachtung Robert E. Kohlers, der argumentiert, dass die Grenze zwischen den Feld- und Laborwissenschaften meist mehr durch kulturelle als durch klar abgrenzbare epistemologische Praktiken gezogen wird (Kohler 2011: 213).

Die Kampagne am Kap Gelidonya lässt sich auch als ein Beispiel der „American Hegemony“ im Kalten Krieg lesen (Krige 2014). Anders als in der Analyse von John Krige ging es hier jedoch nicht um eine von beiden Seiten getragene konsensuale Durchsetzung eines Organisationsprinzips von Wissenschaft. Vielmehr zeigt die Kampagne auf, wie die Verfügbarkeit von Ressourcen in einer Konkurrenzsituation zwischen Forscher*innen und universitären Institutionen zu einer Dominanz von US-Partner*innen führte, die viel besser ausgestattet waren als die noch immer von den Folgen des Zweiten Weltkriegs beeinträchtigten europäischen Kolleg*innen. Frost half in diesem Zusammenhang nicht, dass sie bereits vor dem Start der Kampagne eine Notiz in *Antiquity* untergebracht hatte, die einen ersten Überblick über die Fundorte gab und damit ihren Anspruch am Fundort klar formulierte (Frost 1960). Dieser Text von Frost findet sich nicht in der Bibliografie von Bass’ abschließender Publikation über das „Bronze Wreck“ (Bass 1967a: 7–13). Dass das Zeitalter des europäischen Imperialismus Vergangenheit war, zeigte sich nicht nur daran, dass Frost die Rhetorik und das Auftreten ihres US-amerikanischen Partners zumindest in der Rückschau unerträglich „kolonialistisch“ erschien. Es offenbarte sich auch darin, dass sich nun der Kalte Krieg und nicht länger der Kolonialismus in die Strukturen der Wissenschaft einschrieb. Die konkrete Feldarbeit war somit einerseits von Entwicklungen in der Technik-, Wissens-, und Umweltgeschichte geprägt wie von der Politik- und Zeitgeschichte andererseits.

Frost und Bass beendeten ihre Auseinandersetzung schließlich, als Bass 1972 an Frost schrieb, da er sie mit einem Doktoranden bekannt machen wollte: „It seems to me that 12 years is long enough for a feud to last. I would hope that we might end it.“ Eine Entschuldigung erfolgte nicht, Bass meinte vielmehr: „It would probably not now serve as useful purpose to try understand how it started.“ Seine Publikation zur Kampagne sei mittlerweile erfolgt, er sei zufrieden mit seiner Arbeit und hoffe, dass Frost das auch so sehe. Zugleich lobte er Frosts Arbeit zu Steinankern und an punischen Wracks in Italien. „I do hope that we can bury the axe. It can no longer lead to anything constructive.“^25^ Frost antwortete am 25. November 1972, dass der Doktorand sich gerne bei ihr melden könne und willigte ein, das Kriegsbeil zu begraben: „I quite agree that we can bury one hatchett […] especially as we are both occupied in excavating so many other things including hatchetts!“^26^

## Danksagung

Ich danke der Gerda Henkel Stiftung für die finanzielle Unterstützung, die diesen Aufsatz maßgeblich ermöglicht hat.

## Footnotes

1 Vgl. du Plat Taylor (1965); Basch (1972); Flatman & Staniforth (2006); Bass (2012); Mainberger & Weski (2014); Meide (2013).

2 https://specialcollectionsuniversityofsouthampton.wordpress.com/2018/01/19/honor-frost-archive-weighs-anchor-in-southampton/Zugegriffen: 5. Februar 2021.

3 Nach Evills Tod 1963 erbte Frost sein Vermögen. Neben Kunstwerken gehörte dazu auch ein geräumiges Haus in bester Londoner Innenstadtlage. Nach ihrem Tod 2010 im Alter von 91 Jahren wurde die Kunstsammlung für über 42 Millionen Pfund verkauft. Das wurde das Gründungskapital der Honor Frost Foundation, die der Förderung der Unterwasserarchäologie gewidmet ist (vgl. u. a. den Nachruf im *Guardian* vom 26. Oktober 2010).

4 Einen Ausgangspunkt für ein solches Vorhaben bietet die eher journalistisch gehaltene Arbeit von Faith Warn (2000).

5 „Badeschwamm“, in: *Meyers Konversations-Lexikon*, Band 2., Leipzig: Verlag d. Bibliographischen Instituts 1885, 248–249.

6 „Badeschwamm“, in: *Meyers Großes Konversations-Lexikon*, Band 2., Leipzig: Verlag d. Bibliographischen Instituts 1905, S. 258–259.

7 Zu den frühen unterwasserarchäologischen Grabungen vgl. auch Basch (1972),12–14.

8 Eine historisch-kritische Arbeit zu Cousteau fehlt bislang.

9 Dumas an Frost, Sanary-Sur-Mer, 25. November 1959, University of Southampton Honor-Frost-Archive UoS HFA/5/5, Correspondence with Frédéric Dumas.

10 UoS HFA/5/5, Correspondence with Frédéric Dumas.

11 Projektentwurf: „Project for Underwater Archaeology off the Turkish Coast“ (ohne Ort und Datum, wahrscheinlich vom Jahreswechsel 1959/1960), UoS HFA Bronze & Yassi Ada Wrecks, Preparatory Work, 1958–1960.

12 Ebd.

13 Vorbereitende Notizen und unsortierte Transkripte einer BBC-Radio-Diskussionsrunde vom 3. Januar 1961, UoS HFA/6/1/3 HF Lectures, Various.

14 Ebd.

15 Ebd.

16 Ebd.

17 Ebd.

18 Ebd.

19 Ebd.

20 Dumas an Frost, Sanary-Sur-Mer, 14. März 1961, UoS HFA/5/5, Correspondence with Frédéric Dumas.

21 Ebd.

22 Throckmorton an Frost, Paralos, Mykonos, 13. November 1960, UoS HFA/1/13/3, Correspondence Turkey, c. 1958–1962, 1972, 2000 & undated.

23 http://www.bodrum-museum.com/history-of-the-museum/ (15.04.2019).

24 Dumas an Frost, Sanary-Sur-Mer, 20. Juni 1961, UoS HFA/5/5, Correspondence with Frédéric Dumas.

25 Bass an Frost, Pennsylvania, 28. Oktober 1972, UoS HFA/1/13/3, Correspondence Turkey, c. 1958–1962, 1972, 2000 & undated, Mappe 1972 & 2000.

26 Frost an Bass (ohne Ort), 25. November 1972, UoS HFA/1/13/3, Correspondence Turkey, c. 1958–1962, 1972, 2000 & undated, Mappe 1972 & 2000.
